# Examining how emotional state, tolerance and closeness to others mediate the relationship between closeness to families and empathy in Chinese students

**DOI:** 10.3389/fpsyg.2026.1723348

**Published:** 2026-05-13

**Authors:** Gewei Zhu, Amanda Wayne Madzokere, Yi Zhang, Yanjun Zhang

**Affiliations:** 1 UNESCO Dar es Salaam Office, Dar es Salaam, Tanzania; 2 College of Education, Zhejiang Normal University, Jinhua, Zhejiang, China; 3 Center for European Economics and Finance, Zhejiang Financial College, Hangzhou, Zhejiang, China; 4 Confucius Institute of UDSM, Dar es Salaam, Tanzania

**Keywords:** closeness to families, empathy, multiple mediating effects, SEM, Survey on Social and Emotional Skills

## Abstract

**Introduction:**

In the unique socio-cultural landscape of contemporary China, there is an ongoing scholarly debate regarding whether traditional demandingness or modern responsiveness—reflected as family closeness—is more conducive to fostering social–emotional skills. This study explores the relationship between closeness to families and empathy among students, specifically focusing on the mediating roles of emotional state, tolerance, and closeness to others.

**Methods:**

The study employed a structural equation modeling (SEM)-based mediation analysis using data from the Organization for Economic Co-operation and Development’s (OECD) 2019 Survey on Social and Emotional Skills (SSES). The participants included 3,578 10-year-old students from 76 schools in Suzhou, China.

**Results:**

The results indicate that closeness to families has significant effects on the empathy of students. Furthermore, this effect is completely mediated by three factors: emotional state, tolerance, and closeness to others.

**Discussion:**

The findings underscore that in the modern Chinese context, high levels of responsiveness (family closeness) are essential for students to internalize social norms and develop empathic orientations. The study provides explanations for divergences in previous research through the mediation model and multi-group analysis, highlighting the significant impact of social culture and family education on empathy.

## Introduction

This study aims to investigate the mechanisms through which family closeness influences the empathy of students in Suzhou, China. This chapter first reviewed the research background of empathy, followed by an analysis of the role of family factors, and finally, proposed a theoretical framework integrated with the Chinese cultural context.

### Empathy: a core social–emotional competency

Social and emotional competence refers to a core set of competencies related to self-regulation and social development that adolescents acquire and apply (Osher et al., 2016) and is an important area for adolescents to develop their non-cognitive ability (OECD, 2015). Empathy, defined as the ability to perceive others’ emotional states and generate congruent emotional responses (Decety et al., 2016), is a fundamental pillar of human social development. It serves as a powerful negative predictor of aggressive behavior (Li et al., 2015) and a primary driver of pro-social behaviors, enabling adolescents to better adapt to group life and social interactions. Beyond immediate behavioral outcomes, empathy in adolescence is a robust longitudinal predictor of social competence in adulthood (Allemand et al., 2015). Consequently, fostering empathy has become a global priority in education, as evidenced by major initiatives such as the Social–Emotional Learning (SEL) program in the United States (CASEL, 2026), the SEAL program in the United Kingdom, and the collaborative SEL projects between the Chinese Ministry of Education and UNICEF since 2011.

Despite these efforts, the development of empathy remains uneven across different regions and cultures. For instance, while the OECD (2019) SSES (SSES) provided a global benchmark for these competencies (OECD, 2021), it also revealed significant variations. Notably, students in Suzhou, China, achieved the highest empathy scores among all participating international cities (Daily, 2021). While these results highlight the success of Chinese students in this domain, the specific mechanisms—particularly the role of family dynamics—that contribute to such high levels of empathy remain insufficiently explored in the existing literature.

### Family closeness: the role of family and the need for mechanistic understanding

A critical factor in the development of empathy is the quality of the family environment. From a theoretical perspective, family closeness reflects the dimension of parental responsiveness—the warmth and emotional support that allow children to internalize social norms and develop an orientation toward others (Baumrind, 1991; Alcaide et al., 2025a). While international literature consistently identifies family closeness as a positive predictor of empathy, this relationship is particularly salient in the Chinese context. Analysis of the SSES2019 data suggests that the positive effect of family closeness on empathy was more pronounced in Suzhou than in other participating cities like Helsinki, Istanbul, or Houston.

However, the black box of how family closeness translates into empathy requires further investigation. Current research suggests that this relationship is likely not direct but is mediated by the individual’s internal psychological state and social perceptions. For instance, a strong family self-concept—the sense of being valued within the home—may foster emotional wellbeing and *tolerance,* which in turn facilitate a greater *closeness to others* (social self-concept) and ultimately, higher empathy (Ertema et al., 2025). The distinction between *closeness to family* and closeness to others is rooted in the multi-dimensional nature of an individual’s self-concept. Specifically, these constructs align with the hierarchical model of self-concept proposed by Shavelson et al. (1976), which differentiates between specific domains of self-perception. In this study, closeness to family represents the family self-concept—the extent to which individuals feel loved and appreciated within their domestic sphere—whereas *closeness to others* pertains to the social self-concept, reflecting their perceived quality of peer and extra-familial interactions (Ertema et al., 2025). Recognizing these as distinct psychological pillars is essential for understanding how internal family dynamics translate into external social adjustment.

To date, no study has utilized the comprehensive SSES2019 dataset to model these specific mediating paths for Chinese students. Therefore, the present study aims to examine how emotional wellbeing, tolerance, and closeness to others mediate the relationship between family ties and empathy in Suzhou, China. By elucidating these internal mechanisms, this research seeks to provide evidence-based insights for improving students’ empathy both in China and globally.

### Cultural significance: empathy and family in the Chinese context

To understand the necessity of this research, one must consider the deep-seated cultural underpinnings of empathy and family ties in China. Unlike the Western emphasis on individual autonomy, Chinese culture is traditionally rooted in Confucianism, which defines the self as a *relational self* rather than an isolated entity.

First, empathy is the psychological manifestation of *Ren* (仁-Benevolence), the core virtue of Confucian ethics. Ren begins with the ability to put oneself in another’s shoes (*Shu* - 恕), making empathy a fundamental social obligation for maintaining interpersonal harmony (*He*-和). Consequently, fostering empathy in adolescents is not merely about personal development but about preserving the moral fabric of society.

Second, the importance of family ties is anchored in the concept of *Xiao* (孝 - Filial Piety). In the Chinese context, the family is the primary socialization agent where an individual first learns to navigate social hierarchies and emotional reciprocity. Strong family ties (closeness) act as the *secure base* from which Chinese students extend their emotional orientation toward others.

However, China is currently undergoing a significant transition. While traditional parenting often emphasized *Guan* (管 - to govern/control), which literally means “to govern” and “to love” as Chao (1994) argued, modern Chinese Generation Z students are increasingly seeking emotional responsiveness within these tight family structures. This creates a unique cultural tension: how can traditional family centralism be integrated with modern psychological needs for warmth? Investigating the mechanisms by which family closeness fosters empathy is therefore significant, as it addresses how a culture of relational self-adapts to the demands of modern social–emotional intelligence. This research is necessary to provide a culturally sensitive model for educational interventions in one of the world’s most rapidly changing societies.

### Theoretical framework: parental responsiveness and demandingness

The conceptualization of family dynamics and their impact on child development is fundamentally anchored in the two-dimensional model of parenting socialization (Baumrind, 1991; Maccoby and Martin, 1983). According to this framework, parenting practices are defined by two theoretically orthogonal, or unrelated, dimensions: responsiveness and demandingness (Alcaide et al., 2025a; Jacome-Mora et al., 2025). Responsiveness refers to the extent to which parents foster individuality, self-regulation, and self-assertion by being supportive and attuned to the child’s emotional needs (Baumrind, 1991; Alcaide et al., 2025b). In contrast, demandingness concerns the claims parents make on children to become integrated into the family whole, through supervision, disciplinary efforts, and expectations for maturity (Garcia et al., 2024; Steinberg et al., 1994). Within this theoretical landscape, the focal variable of the present study—closeness to family—serves as a primary indicator of the *responsiveness* dimension. It reflects the emotional warmth, trust, and openness within the family structure that are essential for the healthy development of social skills. By situating family closeness within this responsiveness-demandingness framework, we can more precisely examine how its effects on student empathy are mediated by psychological states and social orientations, particularly within the unique cultural context of China (Garcia et al., 2019).

While the broad benefits of social and emotional competencies for academic and mental health outcomes are well-documented (e.g., Durlak et al., 2011; Taylor et al., 2017), the specific pathways through which these competencies develop within high-performing cohorts remain less clear. The exceptional empathy scores observed in Suzhou students (OECD, 2021) provide a unique opportunity to move beyond general outcome studies and instead investigate the internal psychological and social drivers of these skills. Given the central role of the family in Chinese developmental ecology, there is a critical need to deconstruct how domestic emotional bonds translate into outward social empathy. Therefore, the following literature review examines the existing empirical evidence regarding family responsiveness and its potential mediators to establish the basis for our hypothesized model.

### Literature review and hypotheses

Following the rationale established in the introduction, this chapter reviews empirical evidence linking family closeness to adolescent empathy via parental responsiveness to address the need for a mechanistic understanding mentioned above. We specifically examine how emotional states, social self-concepts, and tolerance mediate this relationship to provide the empirical foundation for our hypothesized model. This synthesis bridges the theoretical framework with our specific analysis of the Chinese developmental context.

### Pro-social behavioral concept

The Pro-social behavioral concept is described as involuntary behavioral characteristics that intend to benefit others when shared or exhibited (Eisenberg and Fabes, 1998). Pro-social behavioral traits can be characterized by acts of empathy, kindness, tolerance and other positive reactions toward others. Parenting styles and disciplinary patterns have been attributed to the development of positive pro-social behaviors (Eisenberg and Fabes, 1998). A child learns and develops behavioral skills from his or her family during the process of growth, childhood experiences shape one’s adolescent and adult behavior. A child growing up in a repressive environment is less likely to exhibit good communication skills as they grow. A conducive environment to cultivate pro-social behavioral traits must be created and nurtured within a family structure. Millet et al. posit that individuals with stronger family structures are more likely to develop and exhibit positive pro-social traits (Miller et al., 2000). A good quality family relationship between a child and parents has significant impact on learned and shared behavior.

Family closeness is a trait and or characteristic of a strong emotionally balanced family function. In the field of developmental psychology, these family dynamics are formally conceptualized through two primary dimensions: responsiveness (warmth and support) and demandingness (strictness and supervision) (Baumrind, 1991; Maccoby and Martin, 1983). Family closeness, as examined in this study, primarily reflects the responsiveness dimension, which has been consistently linked to positive pro-social outcomes in Western contexts. Olson et al. describe family function as effectiveness of various functions of a family structure (Olson et al., 1979). Family communication and family emotional connection are integral characteristics of family function activities that ultimately nature positive traits of pro-social behavior. He goes on to anchor good and effective communication between a child and parents or siblings as a variant to mold a good relationship within the family structure. Emotional connection can be described as an awareness of understanding, showing empathy, trust, and mutual respect, It allows individuals to feel seen, heard, and valued, fostering and creating a sense of closeness and intimacy. A solid emotional connection means a good cultivation of traits and characteristics of pro-social behaviors. An emotionally stable individual can easily exhibit traits of empathy, tolerance, kindness and other positive characteristics. Abdel-Fattah (2020) describes emotional stability as having the capability to enable a person to develop an integrated and balanced way of perceiving the problems of life. The balance ensures and plays a role in nurturing positive emotions and establish that one has traits of tolerance, empathy and logic emotion. A tolerant, empathetic and emotionally stable child is evidence of a strong family function which is a characteristic of family closeness.

In summary, this research will explore and hypothesize how different effects of closeness to family inculcate Pro-social behavioral characteristics exhibited toward others by Chinese students. The mediating effects of closeness to family, emotional state, tolerance and closeness to others are explored to investigate their role in shaping quality social skills in Chinese students.

#### Effects of closeness to families on empathy

The closeness individuals perceive with family members is a core component of the family self-concept, which is theoretically distinct from the social self-concept (Shavelson et al., 1976; Ertema et al., 2025). In SSES2019, some of the survey questions concern the closeness of adolescents to family members, which is the “closeness to families.” The main concepts related to this in the academic community are family closeness, parent–child relationship, and sibling relationship. Empathy refers to the ability of individuals to perceive the emotional state of others in social interactions and to generate similar emotional feelings (Decety et al., 2016). OECD defines it as “Perspective taking and empathic concern for others’ wellbeing” (OECD, 2019). At the microsystem level, broader cultural values and educational policies in China-such as collectivism, Confucian ethics emphasizing ren a.i. (benevolence) and tui ji ji ren (putting oneself in others’ shoes), and an exam-oriented culture- create a unique backdrop against which empathy develops.

Closeness to families refers to the degree of emotional connection individuals perceive with family members, and is an indicator of relationships between families (Liu et al., 2014). Empirical studies for Chinese adolescents have shown that closeness to families can positively predict the development of adolescent empathy, and it is significantly and positively related to their cognitive empathy (Deng et al., 2018; Wang et al., 2019).

Parent–child relationship generally refers to the emotional connection between parent and offspring, and the higher the degree of emotional connection between parents and children, the better the development of adolescent empathy (Acar et al., 2018). Chen and Zhou (2015) demonstrated that strong parent–child relationships are positively associated with the development of children’s empathic ability. Sibling relationships are one of the longest-lasting relationships in life and have an important impact on individual development (Noller, 2005). Lam et al. (2012) conducted a study on sibling relationships and their empathy development in adolescents aged 7–14 years old over 2 years and showed that adolescents with better sibling relationships had better empathy development.

Zhang et al. (2019) found that sibling closeness serves as a significant positive predictor of children’s empathic ability. However, the impact of parenting styles exhibits significant cultural variability. While “strictness without warmth” (authoritarian style) is typically maladaptive in the West, some research suggests it may correlate with academic achievement in Chinese families due to the cultural notion of “Guan” (Chao, 1994). Nevertheless, recent evidence from Chinese Generation Z populations indicates that responsiveness-based parenting (authoritative or indulgent) remains superior for developing emotional intelligence and empathy (Alcaide et al., 2025a; Chen et al., 2024), justifying our hypothesis on the positive role of family closeness.

In summary, it can be seen that adolescents’ closeness to their families is closely related to their empathic ability. This leads to the following hypothetical proposal:

*H1*: Closeness to families has a positive effect on empathy.

#### The mediating role of emotional states

Emotion is “the individual’s psychological experience of whether something objective meets subjective needs” (Li, 2008), and an emotional state is a certain emotion produced by an individual under the influence of a certain event or situation in a certain period (Ye, 2001).

High-quality family and interpersonal relationships tend to promote positive emotions (Kwok et al., 2020). Fosco et al. (2012) suggest that living in a highly intimate family atmosphere for a long time can promote the development of life adjustment skills and reduce the risk of negative emotional distress in adolescents. Several empirical studies conducted in China have similarly shown that good family and parent–child relationships can have a significant positive effect on students’ sense of efficacy in emotion regulation, and that closeness to family helps maintain good emotional states in adolescents (Huang et al., 2015; Wang et al., 2016; Wang et al., 2017). In addition, studies oriented to Chinese left-behind children have also demonstrated that closeness to family members is significantly positively related to their positive emotions and significantly negatively related to negative emotions (Zhao et al., 2017).

Emotional states can affect adolescents’ empathic performance. When individuals are in a positive emotional state, they are more likely to focus on the needs of others and understand their emotions more positively and constructively. In contrast, when individuals are in a negative emotional state, they are more focused on their own needs or are unable to concentrate due to the negative influence, which can weaken empathy (Staub, 1984; Davis, 1994; Li et al., 2018). Emotion regulation ability is important for individuals to maintain positive emotional states. Adolescents with high emotion regulation ability can use emotion regulation strategies to effectively manage negative emotions to maintain their positive emotional states (Panfile and Laible, 2012; Hein et al., 2018; Benita et al., 2017). Research has shown a significant positive correlation between adolescents’ emotion regulation strategies, empathic ability, and pro-social behavior (Yu, 2021). In contrast, some studies have also shown that negative emotions such as sadness and anger can also increase individuals’ empathic and helpful behaviors (Baumann et al., 1981; Yuan et al., 2021).

Based on the perspective that high-quality family relationships may lead to positive emotional states and positive emotional states may positively influence adolescents’ empathic abilities, this study proposed the hypothesis 2:

*H2*: Emotional states play a mediating role in the effect of closeness to families on empathy.

#### The mediating role of closeness to others

While family closeness shapes the internal self-view, closeness to others represents the social self-concept. Recent evidence suggests that these two constructs have distinct relationships with personal adjustment and social orientation (Ertema et al., 2025). For elementary school students, peer interactions are a major component of their social interactions (Ruan, 2004). Peer relationships refer to a type of interpersonal relationship established and developed in the process of interaction between peers or between individuals at comparable levels of psychological development (Zhou, 1998).

Studies have pointed out that families with a high level of closeness give more support and love to adolescents, which is beneficial to their development in many aspects. At the same time, adolescents will consciously imitate the good ways and behaviors of their family members (Liu and Wang, 2005). Leidy et al. (2010) studied the relationship between Latino immigrant family closeness and social competence, it was found that adolescents with higher family closeness had better social skills. Tan et al. (2015) identified parent–child relationships as an important factor influencing adolescent peer relationships through a survey and found that adolescents’ parent–child relationships significantly reduced levels of relational aggression and thus formed cordial social relationships with peers. In a slight difference from the above studies, Romig and Bakken (1992) found gender differences in the association between family closeness and closeness to others, and they concluded that family closeness positively and significantly predicted girls’ closeness to others, but could not predict boys’. Yang (2019) also noted that girls’ levels of healthy dating and altruistic behaviors were significantly higher than boys’ under the effect of similar family factors.

As an important component of the family system, sibling relationships are also important for the development of peer relationships, and Smorti and Ponti (2018) showed that the quality of sibling relationships can positively influence the quality of peer relationships through the mediation of students’ prosocial behavior. In China, several empirical studies with children, their parents, and teachers have shown that sibling relationships have a significant positive predictive effect on adolescents’ pro-social behavior and that children with closer sibling relationships have better social skills, which in turn influence their close relationships with peers (Gao et al., 2021; Zheng, 2022). A study confirmed this view from the opposite direction. This study showed that among adolescents in Shanxi Province, China, sibling conflict positively and significantly predicted poor peer interactions among middle school students (Zhang et al., 2020). Similarly, evidence suggests that closer sibling relationships promote positive peer interactions (Lv, 2021).

The quality of relationships with others reflects whether individuals have good interaction strategies and methods, which is extremely important for the development of empathy. A meta-analysis involving 70 studies on the relationship between the quality of parent–child relationships, the quality of peer relationships, and adolescent empathy noted that peer relationships, parent–child relationships, and adolescent empathy all showed significant positive correlations, while the correlation between peer relationships and adolescent empathy was significantly higher than that between parent–child relationships and adolescent empathy, which had a more direct effect on adolescent empathy (Boele et al., 2019). The results of an observation and questionnaire study of 623 elementary school students in Fujian Province, China, showed that peer relationships are an important factor that can strongly influence empathy. Besides, the closeness of peer relationships affects the empathic ability of individuals, and close peer relationships make students more likely to develop empathic responses to others (Chen, 2020).

Based on the relationships among high-level family closeness, healthy and close relationship to others, and empathy, we proposed the hypothesis 3:

*H3*: Closeness to others play a mediating role in the effect of closeness to families on empathy.

#### The mediating role of tolerance

One of the fundamental features of modern education is the agglomeration of peers and the compulsory interaction between peers from all social strata, which means that tolerance will be a necessary quality for them to live together (Gao, 2020). The UNESCO Declaration of Principles on Toleration defines tolerance as “respect, acceptance, and appreciation of the rich diversity of the world’s cultures, their different forms of expression, their ways of being” (UNESCO, 1996). The OECD defines it as “Being open to different points of view, values diversity, is appreciative of foreign people and cultures” (OECD, 2019). Chinese scholars’ studies on interpersonal relationships point out that interpersonal emotions are closely related to adolescents’ academic performance, interpersonal interactions, and physical and mental health, and that tolerance is an important component of interpersonal emotions (Lu, 2009). Although there are various definitions of tolerance, all of them have the connotation of going out of oneself and toward others, which is in line with empathy, that is, only by going toward others can one put oneself in the shoes of others, consider others and thus empathize with them.

Studies on family relationships and Chinese students’ tolerance ability show that a good family atmosphere helps adolescents to develop tolerance. Song (2006) pointed out that friendship and tolerance among family members can create a harmonious and friendly family environment, and adolescents will gradually develop good qualities of tolerance in this environment. In contrast, a poor family atmosphere is not conducive to the development of tolerance in adolescents and makes them more likely to behave negatively when dealing with interpersonal relationships. Longitudinal evidence indicates that the development of tolerance is hindered in children exposed to parental marital conflict (Wang, 2020).

**Figure 1:**
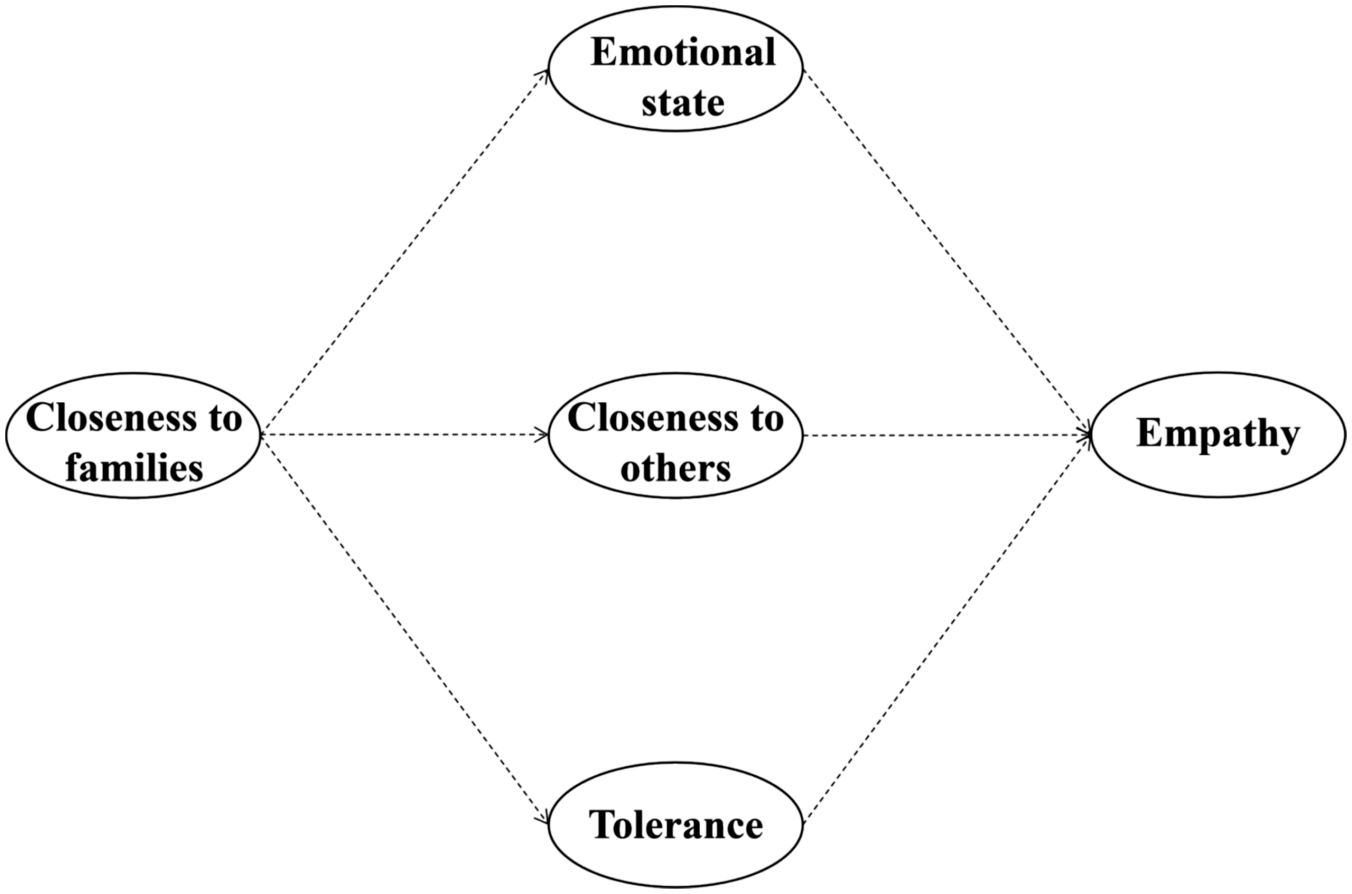
Mediation model linking closeness to families and Empathy.

Some studies have pointed out that tolerance is closely related to understanding and empathizing with others in China. Tolerance is a basic requirement for individuals in modern society. People who have tolerance are more able to overcome their self-centered consciousness and think differently about the people they interact with to understand the situation and position of others (Tang, 2009). From the perspective of traditional Chinese ancient culture, it was pointed out that Confucianism, Taoism, and Mohism, which have the most profound influence on traditional Chinese culture, are important cornerstones of Chinese people’s tolerance mentality. They all require individuals to know how to put themselves in others’ perspective and consider others, to have empathy with others, and to have the ability to empathize with others (Qian and Yu, 2014). It can be seen that both tolerance and closeness to family and empathy show interrelated characteristics. Thus, the following hypothesis is proposed (Figure 1):

*H4*: Tolerance plays a mediating role in the effect of closeness to families on empathy.

By integrating these factors, we propose a holistic model where family closeness (responsiveness) first fosters a stable emotional state and a positive family self-concept. These internal resources then facilitate tolerance and closeness to others (social self-concept), which finally manifest as heightened empathy. This sequential mediation aligns with the recent call for examining how cultural contexts shape the internalization of social norms (Garcia et al., 2019; Ertema et al., 2025).

## Methodology

### Participants

Based on the data published by the SSES 2019 program, to exam the general influence of closeness to families to empathy, all 31,804 10-year-old students participated in SSES 2019 were included (Girls 49.4% and Boys 50.6%). The main subjects of this study were 3,800 primary school students in the 10-year-old group who participated in the OECD Social and Emotional Competence Assessment. They were tested from 76 schools drawn from 10 districts in Suzhou, using a combination of explicit and invisible stratification (OECD, 2021). A total of 3,578 valid data (Girls 45.8% and Boys 54.2%) was obtained with invalid data being eliminated, missing data was all deleted. In this study, Suzhou (China) as a representative, likes Daegu (Korea), and Helsinki (Finland), is globally recognized for their high performance in cognitive assessments (e.g., PISA). Researching SSES in a city like China’s Suzhou is essential to understand the hidden emotional costs and supports behind academic excellence.

### Measure

Based on the *OECD SSES Technical Report* (OECD, 2021), the data employed in this study was obtained through the scientifically rigorous sampling procedures implemented by the OECD SSES 2019 program team. The instruments used, as well as the data collection procedures, are underpinned by robust theoretical frameworks and established quality assurance mechanisms, thereby ensuring the reliability and validity of the dataset.

### Variable selection and processing

#### Closeness to families

The independent variable of the study was the closeness to families, which reflects the closeness of the students to their family members. It consists of four questions in the student questionnaire: “How close to your mother,” “How close to your father,” “How close to your brothers and sisters,” and “How close to your relatives.” The options ranged from 1 to 5, from Not at all to Very close.

#### Empathy

The dependent variable in this study was empathy, which means “Understands and cares about others, and their wellbeing. Values and invests in close relationships.” Empathy is a sub-dimension of the collaboration dimension in the theoretical framework of SSES 2019. The options ranged from 1 to 5, from Strongly Disagree to Strongly Agree, after reversing the reverse questions.

#### Tolerance, closeness to others and emotional state

The three mediating variables involved in the study were tolerance, closeness to others, and emotional state. As for tolerance, it means “Is open to different points of view, values diversity, is appreciative of foreign people and cultures,” which belongs to the open-mindedness dimension in the theoretical framework of SSES2019. Closeness to others consists of four questions from the student questionnaire, namely “How close to your friends, How close to your classmates, How close to your favorite teacher, and How close to your neighbor.” The emotional state consisted of the questions “Last two weeks: felt cheerful and in good spirits, Last two weeks: felt calm and relaxed, Last two weeks: felt active and vigorous, Last two weeks: woken up feeling fresh and rested and Last two weeks: daily life filled with things that interest me.” The above three variables correspond to questions with options ranging from 1 to 5, like “At no time” to “All of the time.”

### Validity and reliability testing

Table 1 presents the final Confirmatory Factor Analysis (CFA) results, The Non-standardized estimation parameters of items for all measurement models were all positive and significant, and the standardization coefficient were all higher than 0.5, Squared Multiple Correlation (SMC) were all higher than 0.25, and Composite Reliability (CR) values were all higher than 0.7. Since all indicators were better than the acceptable values, it means the measurement indicators reflected the characteristics of the latent variables, and the model was found to have good structural validity. According to the suggestion of Fornell and Larcker (1981) the value of the Average Variance Extracted (AVE) is greater than 0.36 acceptable, Therefore individual variables have sufficient convergent validity. As shown in Table 2, the pearson correlation coefficients between the variables are smaller than the value of the square root of the AVE on the diagonal, which proves the existence of differential validity between the variables. The model fit indicators are also satisfactory (CMNI/df = 1.16 GFI = 0.96 CFI = 0.96 AGFI = 0.95 RMSEA = 0.41).

**Table 1:** Results of CFA.

Items	Unstd.	Std.	S. E.	SMC	CR	AVE
Closeness to families						
Closeness to mother	1.000	0.698		0.487	0.785	0.479
Closeness to father	1.239	0.770	0.034	0.593		
Closeness to brothers and sisters	1.175	0.649	0.036	0.421		
Closeness to relatives	1.006	0.644	0.031	0.415		
Empathy						
EMP01	1.000	0.585		0.342	0.788	0.427
EMP03	1.074	0.632	0.038	0.399		
EMP04	1.173	0.634	0.041	0.402		
EMP06	1.312	0.715	0.043	0.511		
EMP07	1.214	0.694	0.040	0.482		
Emotional state						
Felt cheerful and in good spirits	1.000	0.781		0.610	0.865	0.563
Felt calm and relaxed	0.981	0.754	0.022	0.569		
Felt active and vigorous	1.005	0.731	0.023	0.534		
Woken up feeling fresh and rested	1.173	0.749	0.026	0.561		
Daily life filled with things that interest me	0.984	0.735	0.022	0.540		
Tolerance						
TOL02	1.000	0.598		0.358	0.752	0.435
TOL05	1.272	0.676	0.043	0.457		
TOL06	1.058	0.567	0.041	0.321		
TOL08	1.380	0.776	0.044	0.602		
Closeness to others						
Closeness to friends	1.000	0.815		0.664	0.840	0.573
Closeness to classmates	1.144	0.865	0.021	0.748		
Closeness to favorite teacher	0.992	0.734	0.022	0.539		
Closeness to neighbors	0.888	0.584	0.025	0.341		

**Table 2:** Correlation and differential validity between variables.

	1	2	3	4	5
1	0.692				
2	0.363	0.653			
3	0.441	0.574	0.750		
4	0.273	0.634	0.445	0.660	
5	0.560	0.399	0.396	0.288	0.757
M	4.247	4.194	3.598	3.998	3.384
SD	0.847	0.636	0.921	0.782	0.995

1: Closeness to families; 2: Empathy; 3: Emotional state; 4: Tolerance; 5: Closeness to others; M, Mean; SD, Standard deviation.

### Analysis

#### Regression analysis

The main objective was to use regression analysis to explore whether there were some commonalities among the students participating in SSES 2019 and to build on these commonalities to develop a study with broader benefits. Specifically, whether the closeness to families had a significant effect on the empathy of all urban elementary school students in each country that participated in the survey. During this period, Amos 24.0 was used.

#### Mediation effects testing

The purpose of the mediating effect analysis was to investigate the internal mechanism of closeness to families on students’ empathy and to find more variables that could directly influence students’ empathy while providing insight into why Chinese students in Suzhou excel in empathy. Based on the Literature Review, it was assumed that closeness to others, emotional state, and tolerance were the main mediating variables in this study. It was hoped that understanding their roles in the effect of closeness to families on empathy and making a comparison of the importance of each mediating pathway could help to further strengthen the empathic ability of students in China as well as provide some practicing ways to enhance the same ability of students in the same age globally. Amos 24.0 was used for mediation effects testing.

## Results

### Closeness to families positively and significantly affected empathy in all countries’ 10-year-old groups and was highest in China

The outcomes of regression analysis about all students’ closeness to families on empathy are shown in Table 3. As can be seen, closeness to families positively and significantly influenced the empathy of elementary school students in the 10-year-old group in all cities participating in the assessment, hypotheses 1 was verified. However, the most obvious degree of influence was found in the Suzhou group in China, as shown in Table 3, it was significantly higher than the average.

**Table 3:** Effect of closeness to families on empathy (Suzhou compared with all cities).

Group	Estimate	S. E.	*Z*	Bias-corrected		Percentile		*P*
				Lower	Upper	Lower	Upper	
All cities	0.163	0.006	27.167	0.152	0.176	0.151	0.176	***
Suzhou	0.270	0.021	12.857	0.229	0.312	0.229	0.311	***
Suzhou compared with all	0.106	0.022	4.818	0.064	0.149	0.063	0.149	***

***Indicates *p* < 0.001.

### Fully mediated effects of emotional state, tolerance, and closeness to others

The study used structural equation modeling to test for mediating effects through the bias-corrected non-parametric percentile Bootstrap method, as suggested by Fang et al. (2014). The 95% confidence intervals were recorded after 5,000 repetitions of sampling, and then Z and *p*-values were calculated based on the point estimates as well as standard error. If the absolute value of the *Z*-value is greater than 1.96 and the *p*-value is significant and the 95% confidence interval does not contain 0, the total, direct, or mediating effect is valid.

Through the analysis of the data, the study found that the direct effect was not significant in the relationship between the effect of closeness to families on empathy for the 10-year-old group of students in Suzhou, China, as shown in Table 4. Because the direct effects were not significant, the study will present the results of the analysis of the mediating and total effects separately, as shown in Table 5.

**Table 4:** Direct effect tests.

Path	Estimate	S. E.	*Z*	Bias-corrected		Percentile		*P*
				Lower	Upper	Lower	Upper	
Closeness to families→Empathy	−0.008	0.021	−0.381	−0.05	0.035	−0.049	0.035	0.703

**Table 5:** Tests for medicating effects.

Path	Estimate	S. E.	*Z*	Bias-corrected		Percentile		*P*	Ratio
				Lower	Upper	Lower	Upper		
Indirect path 1	0.126	0.012	10.500	0.105	0.152	0.104	0.150	***	41.59%
Indirect path 2	0.112	0.012	9.333	0.090	0.138	0.089	0.137	***	36.96%
Indirect path 3	0.065	0.009	7.222	0.048	0.085	0.047	0.084	***	21.45%
Total path	0.303	0.023	13.174	0.261	0.353	0.260	0.351	***	

***Indicates *p* < 0.001; Indirect Path 1: Closeness to families→Emotional state→Empathy; Indirect Path 2: Closeness to families→Tolerance→ Empathy; Indirect Path 3: Closeness to families→Closeness to others→Empathy; Total Path: Total Closeness to families→Empathy.

**Figure 2:**
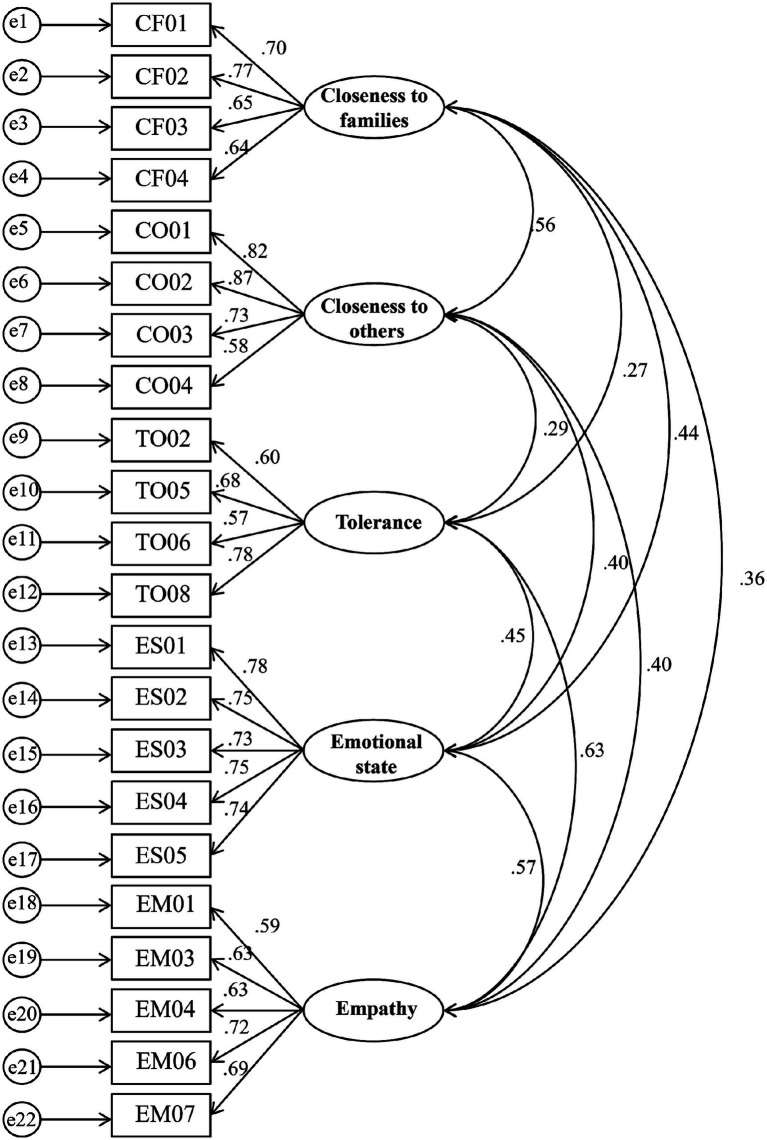
CFA model with standardized factor loadings.

**Figure 3:**
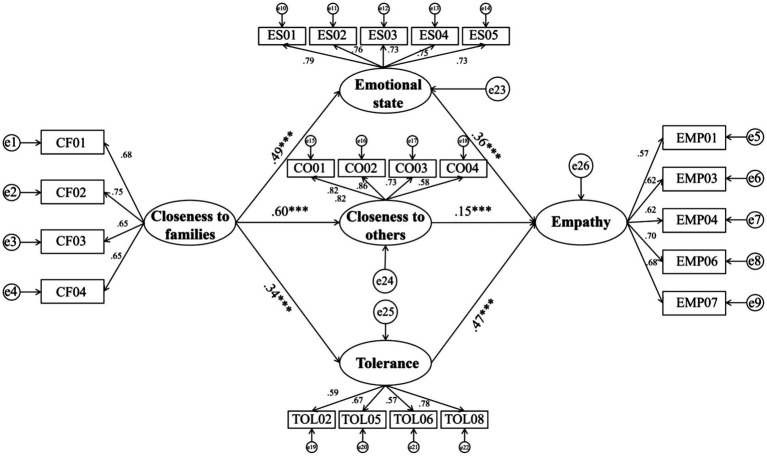
Mediation model linking closeness to families and Empathy.

From the results of the data analysis, the three mediating variables emotional state, tolerance, and closeness to others played a fully mediating role in the effect of closeness to family on students’ empathy, constituting a multiple mediation model, as shown in Figure 2, the model fit was acceptable (CMIN/df = 1.26, GFI = 0.95, CFI = 0.95, AGFI = 0.94, RMSEA = 0.05). Hypotheses 2, hypotheses 3, and hypotheses 4 were verified. In terms of effect, the emotional state mediating variable played the most significant mediating role (41.59%), followed by the tolerance mediating variable (36.96%), and finally closeness to others mediating variable (21.45%) (Figure 3).

In terms of mediating effects, it was found that closeness to family did not have a direct and significant effect on empathy for Chinese students. It was the emotional state, closeness to others, and tolerance that mediated the relationship between closeness to family members and empathy, and they were fully mediated. The identification of mediating variables implies more directions for interventions to improve students’ empathy, such as developing students’ tolerance, maintaining students’ positive emotional state or closeness to others to improve their empathy when it is not possible to efficiently improve their closeness to family members, which is important for the diversification of educational tools and for This has important implications for the diversification of educational tools and student-centered personalized education. In addition, the discovery of mediating variables can also broaden the ideas of research related to this ability.

## Discussion

### The role of family closeness in fostering empathy

Some studies have shown that the resilience of Chinese national culture still plays a strong role in modern society (Peng, 2014; Wu and Liu, 2022). The existence of commonalities and significant differences highlight the importance of further exploration and explanatory of the internal mechanisms of the effects of closeness to families on empathy, with a focus on Chinese students to provide recommendations for countries around the world.

The family education is highly emphasized in Chinese society and culture. Family and variations in child development depending on culture. In general, studies in the West indicate that the use of demandingness without warmth (authoritarian style) leads to negative outcomes, causing children to feel that their family does not love or value them. In contrast, using demandingness with responsiveness (authoritative style) yields more positive results (Lamborn et al., 1991; Maccoby and Martin, 1983; Steinberg et al., 1994). However, the same parenting practices may have different implications around the world (Garcia et al., 2019; Martinez-Escudero et al., 2023; Pinquart and Gochaliyev, 2025). Specifically, there has been discussion about why the use of demandingness without the component of responsiveness does not always lead to negative outcomes; children may feel protected by the family and loved, and thus do not experience adjustment problems (Ertema et al., 2025; Garcia et al., 2019; Villarejo et al., 2024). Chinese family education mostly demonstrated these cases.

At the traditional cultural level, Chinese society has always been very concerned with raising the next generation’s concept of the family and has been emphasizing the importance of family education. As a pillar of traditional Chinese culture, Confucianism emphasizes the greatness of the family and the state, viewing the individual, the family, the state, and the world as one social continuum, and emphasizing the “family-state homogeneity” (Ma and Liang, 2021). The concept of “family-state homogeneity” believes that building the family and building the state is intrinsically consistent, and the two are mutually complete and supportive. It reflects the idea that the family and the state are one and the individual and the collective coexist in Chinese culture (Xu, 2020). Under its influence, Chinese society has focused on establishing quality family style and maintaining good family relationships from ancient times to the present, resulting in a series of cultural treasures related to family education that are household names. For example, the story of “Mencius’ Mother Moved Three Times,” poems celebrating kinship such as “Song of the Parting Son,” and writings on family discipline and family rules such as “Yan’s Family Discipline.”

In recent years, China has paid more and more attention to cultivating students’ correct family values and has emphasized the important role of family education in the “iron triangle” composed of family, school, and society (Peng and Cao, 2022). Xi Jinping, president of the people’s Republic of China pointed out that “the happiness of the family is closely related to the prosperity of the country” when talking about the relationship between the family and the country (People’s Daily, 2019). In 2015, the Ministry of Education of the People’s Republic of China issued the “Guidance on Strengthening Family Education,” stating that “the family is the basic cell of society” and “family education is the most important element of society” (Ministry of Education of the People’s Republic of China, 2015). In 2016, China established 10 experimental family education zones at different levels nationwide to guide the development of family education (Ministry of Education of the People’s Republic of China, 2017). In 2021, the Law of the People’s Republic of China on the Promotion of Family Education was promulgated. In 2021, the Law of the People’s Republic of China on the Promotion of Family Education was promulgated, and family education was officially elevated from a “family matter” to a “national matter,” with the first article of the law clearly stating that it should “guide the whole society to focus on family, family education, and family style to increase family happiness and social harmony” (Ministry of Education of the People’s Republic of China, 2021). In 2023, the Ministry of Education of the People’s Republic of China and other thirteen departments issued the Opinions on Improving the Cooperative Parenting Mechanism of Schools, Families, and Society, which further emphasized family education and positioned its responsibilities (Ministry of Education of the People’s Republic of China, 2023). Compared with the heterogeneous values of family and nation in the West, which attach more importance to individual rights and the “small self,” it can be seen that the emphasis on family education in Chinese society and the introduction of a series of laws and policies, as well as the social culture that advocates family sentiment and family values, have greatly enhanced the influence of the family in the minds of students and provided a good policy for the family to contribute to the development of empathy among Chinese adolescents. This has provided a good policy guarantee and social atmosphere for families to help Chinese youth develop their empathy.

### The importance of empathy in Chinese family education

Family education, with a parent–child relationship as the link, aims to cultivate qualified social beings and is an important factor influencing youth development (Guan, 1994). Despite various influences within family and academic contexts (Gomez-Ortiz et al., 2025; Lamborn et al., 1991), parenting remains consistently related to differences in child development. Some studies identified benefits related to authoritarian parenting, parents who are strict and overprotective, but not warm (Chao, 2001; Dwairy et al., 2006). Within Chinese American families, authoritarian parenting is related to benefits, especially in academic achievement (Chao, 1994, 2001), but other recent evidence from Chinese families revealed authoritarian as negative parenting and benefits related to parenting based on responsiveness (indulgent and authoritative) for self-concept and self-esteem (Alcaide et al., 2025a; Chen et al., 2024). Overall, it is argued that the cultural context in which parental socialization takes place could explain why the relationship between parenting and child adjustment seems not always to be the same (Garcia et al., 2019; Pinquart and Kauser, 2018). It is possible that the extent to which children feel loved and appreciated by their family (the so-called family self-concept) could explain why children internalize social norms and have a healthy development (i.e., Ertema et al., 2025).

Influenced by Confucianism, Taoism, and other traditional Chinese cultural schools, Chinese family education mostly respects the “culture of harmony” in educational thinking, emphasizing “harmony is precious” and worshiping harmony among people (Zhang, 2006). Parents usually teach their children to think differently and learn to perceive and understand the emotions of others in interpersonal interactions, which promotes the development of empathy in adolescents. As the core philosophical background of Chinese family education culture (Park and Chesla, 2007), the influence of Confucianism on Chinese-style family education is unprecedented in its magnitude and breadth of influence. The spirit of benevolence, which is usually held as the core of family education by Confucianism, is reflected in the friendly relationship between individuals and others and between individuals and society. And its advocacy of putting people first, helping each other, and thinking of others as the basic guidelines for dealing with interpersonal relationships was considered as coinciding with the connotation of empathy (Ding, 2018).

From another perspective, studies have shown that in China, the lack of family education will hinder the development of adolescents’ empathy, and children growing up in such an environment are more likely to exhibit emotional indifference and lag in the development of empathy (Liu and Cui, 2020). It is evident that Chinese family education and its emphasis on empathy may be one of the important reasons for Chinese adolescents’ superior performance in empathy.

Furthermore, our findings regarding the positive impact of family closeness align with the broader theoretical framework of parental responsiveness, a key dimension in the classic parenting model (Baumrind, 1991; Maccoby and Martin, 1983). While traditional Western literature often posits that the authoritative style (high responsiveness combined with high demandingness) is the universal optimum, recent cross-cultural evidence suggests a more nuanced reality. As highlighted by Alcaide et al. (2025a) and Chen et al. (2024) in their studies on Chinese Generation Z and contemporary families, parental warmth and responsiveness—captured in our study as closeness to family—play a pivotal role in fostering healthy psychosocial adjustment, including the development of empathy and self-esteem.

Interestingly, the cultural context of China may re-contextualize the meaning of parental control. While “demandingness” without warmth is traditionally associated with negative outcomes in European-American contexts, recent scholarship (e.g., Garcia et al., 2019; Ertema et al., 2025) suggests that in collectivistic societies, children may perceive parental strictness as a form of protection or “Guan” (training), provided it is anchored in a strong emotional bond. By distinguishing between family and social self-concepts, our results reinforce the idea that the internal sense of being loved and valued within the family (the “family self-concept”) is a prerequisite for students to internalize social norms and develop empathetic orientations toward others (Ertema et al., 2025; Jacome-Mora et al., 2025).

### Positive relationship between emotional states and empathy

Some of the studies that have been conducted on the effect of emotional states on empathy suggested that positive emotional states promote empathy (Staub, 1984; Davis, 1994; Li et al., 2018; Yu, 2021), while others thought that negative emotions could also promote empathy (Baumann et al., 1981; Yuan et al., 2021). The present study showed that in a Chinese elementary school student population, emotional states positively and significantly influenced empathy, as shown in Table 6, which supports the idea that positive emotional states are more likely to be positively associated with empathy. This may be related to the fact that only a portion of negative emotions such as sadness may be associated with empathy, while other negative emotions are not associated with empathy and serve to inhibit empathy production (Yang et al., 2017).

**Table 6:** Path significance test.

Path	Estimate	S. E.	*Z*	Bias-corrected		Percentile		*P*
				Lower	Upper	Lower	Upper	
Emotional state→Empathy	0.204	0.014	14.571	0.176	0.232	0.175	0.231	***
Female: closeness to families→Close to others (*N* = 1,636)	0.839	0.066	12.712	0.721	0.979	0.719	0.979	***
Male: Closeness to families→Close to others (*N* = 1,938)	0.801	0.061	13.131	0.688	0.927	0.686	0.924	***
Compare male to female	−0.038	0.079	−0.481	−0.199	0.115	−0.199	0.115	0.617

***p < 0.001.

### Gender invariance in family-social connections

While some studies have suggested that there is a significant gender difference in the effect of closeness to families on closeness to others (Romig and Bakken, 1992; Yang, 2019), the present study concluded the opposite, that is, closeness to families positively and significantly predicted the relationship between girls and others, and also positively and significantly predicted the relationship between boys and others, and there was no significant gender difference in the relationship between closeness to family and closeness to others, as shown in Table 6. While earlier research often aggregated various social ties into a single construct, recent evidence suggests a distinct relationship between family variables and personal adjustment depending on the self-concept domain (Ertema et al., 2025; Jacome-Mora et al., 2025). The family self-concept acts as an emotional foundation; when students feel securely connected to their family, they develop a stable internal sense of value. This internal security then serves as a prerequisite for the development of a positive social self-concept (closeness to others), as children tend to internalize the healthy interaction patterns learned at home and project them onto their peer relationships. This theoretical distinction provides a clearer logic for our mediation model, suggesting that the path from family ties to empathy is not direct, but is channeled through the reinforcement of different self-concept domains. This may be related to the fact that modern Chinese family education has changed from the old way of treating men and women differently, and the social culture of preference for men over women has gradually dissipated. Data shows that the expected number of years of education for girls in China today has surpassed that of boys, and the number of girls in school also exceeds that of boys (Wu and Liu, 2020). The increasing respect for gender differences in families and society may be an important reason why Chinese students are not affected by gender differences in terms of the relationship between closeness to families and closeness to other persons.

### The “Suzhou model”: SEL integration and educational

According to the OCED 2021 report summary Suzhou’s highest scores in the assessment could be attributed to the following:

#### Economic factors

According to the survey report Suzhou had the lowest level of unemployment amongst the participating countries with less than 2 percent unemployed in the city. This is evidence that the economic status of the participants is relatively good. Suzhou city also had a good record of educational investments. The report states that “Suzhou invests a similar percentage of gross domestic product (GDP) on education as the OECD average, with an estimated 5% of the GDP spent on public and private schooling.” This is evidence in itself that educational system is competent and less stressful on the students as the city has a good educational system.

#### Social and emotional learning in schools

As part of its Primary and Secondary curriculum, China has embedded social and emotional learning into their education goals and curriculum for primary and secondary education. The scope of these courses encompasses emotional attitudes, values and skill to promote not just an academically gifted individual but also a socially competent member of society. Students are encouraged to participate in social cooperate responsibility activities, acts of service and creativity (OECD Report, 2019). According to Suzhou, the educational system and curricula invested in laying a foundation for the health and development of not just academics but family and social lives too. All these traits taught and socialized into the students aim to have an empathetic, tolerant, emotionally stable, kind and other positive vices type of a student.

According to the OECD report all other participating cities unlike Suzhou did not have a formal evaluation of students social and emotional skill at national level. The omission of this evaluation scale means that the schools and various related bodies cannot formally track the growth of social and emotional skills in their students. They may have difficulties in offering solutions to improving or developing these skills in their learners, thus having socially and emotionally deficient learners.

#### The lowest rate of bullying

Research results also showed that China had the lowest rate of bullying. The students rate of healthy co-existing and cooperation was relatively higher in China than the other cities that participated in the survey. Research on bullying has shown that students who get bullied at school suffer from lack of self, esteem, mental health and lower academic grades. It also affects the development of their positive social skills. Wang (2023) posits that Individuals with a history of bullying had a higher likelihood to develop a conduct disorder and an antisocial personality disorder than their non-bullying peers, even after controlling for other variables like family history of antisocial behavior and sociodemographic. Suzhou learners showed that they had lower rates of bullying thus the negative effects of bullying did not affect their social and emotional skills, unlike other students from other cities that participated in the OECD assessment.

### Theoretical and practical implications

The findings of this study offer multi-dimensional insights for academic research, educational practice, and national policy formulation, targeting three specific stakeholder groups:

#### Theoretical implications for international education researchers

This study challenges universalist parenting models by introducing a context-sensitive “emotional foundation” framework. It demonstrates that in Confucian contexts, parental demandingness functions as *Guan* (careful training) rather than coercive control when anchored in family closeness. Furthermore, it provides empirical support for the “family-state homogeneity” construct, bridging micro-level family bonding with macro-level civic virtue.

#### Practical implications for school administrators

School leaders should prioritize Emotional Regulation Training, as positive emotional states are the statistical engine of empathy. We recommend transitioning to an “Iron Triangle” approach where schools coach parents on emotional responsiveness. Additionally, administrators should implement standardized SEL tracking tools to proactively monitor school climate and reduce bullying.

#### Policy implications for policymakers and implementers

This research validates the *Law of the People’s Republic of China on the Promotion of Family Education* (2021). Policymakers should treat family closeness as a strategic social asset. We advocate for the nationalization of the “Suzhou Prototype”—high educational investment combined with embedded SEL curricula—to ensure social–emotional development is treated with institutional rigor.

## Limitations and future research

The limitations of the study is that all data chosen was self-reported by students, which may or may not entirely represent the actual situation. Future studies could include the data of teachers or parents published by the OECD. Second, the current sample focused only on students in Suzhou which is an economically developed city among many cities in China. Therefore, the result may not represent the entire population of students in China.

Future studies can use the tools given by OECD to do the same survey in more cities in China. Third, the current research design of OECD was a cross-sectional study, which means that the result of the research captured the situation only at a specific time. In that case, a longitudinal design would be helpful to better understand this relationship in future studies.

## Conclusion

This study establishes that closeness to family is the fundamental “cell” for fostering empathy among Chinese adolescents. By examining the mediating role of emotional states and the cultural framework of “family-state homogeneity,” we have demonstrated that a warm, responsive family environment provides the psychological security necessary for students to internalize social norms and engage in prosocial behavior. The success of the “Suzhou Model” illustrates that when traditional family values are supported by robust educational investment and formalized SEL policies, it results in an ecosystem characterized by high empathy and minimal bullying. Ultimately, these findings underscore that the path to a harmonious society begins with the emotional bond between the individual and the family, providing a viable blueprint for educational systems worldwide.

## Data Availability

The data that support the findings of this study are available from the corresponding author upon reasonable request.
